# Construction of a genome-wide genetic linkage map and identification of quantitative trait loci for powdery mildew resistance in *Gerbera* daisy

**DOI:** 10.3389/fpls.2022.1072717

**Published:** 2023-01-06

**Authors:** Krishna Bhattarai, Sadikshya Sharma, Sujeet Verma, Natalia A. Peres, Shunyuan Xiao, David G. Clark, Zhanao Deng

**Affiliations:** ^1^ Department of Environmental Horticulture, Gulf Coast Research and Education Center, University of Florida, Institute of Food and Agricultural Sciences (IFAS), Wimauma, FL, United States; ^2^ Department of Horticultural Science, Gulf Coast Research and Education Center, University of Florida, Wimauma, FL, United States; ^3^ Department of Plant Pathology, Gulf Coast Research and Education Center, University of Florida, IFAS, Wimauma, FL, United States; ^4^ University of Maryland, Institute for Bioscience and Biotechnology Research, University of Maryland College Park, Rockville, MD, United States; ^5^ Department of Environmental Horticulture, University of Florida, IFAS, Gainesville, FL, United States

**Keywords:** *Gerbera (Gerbera hybrida)*, powdery mildew resistance, genetic linkage map, QTL, disease resistance QTL, genotyping by sequecncing, single nucleotide polymorphism

## Abstract

Powdery mildew (PM) is a common fungal disease in many important crops. The PM caused by *Podosphaera xanthii* has been the most challenging problem in commercial *Gerbera* (*Gerbera hybrida*) production globally, often leading to severe losses of crop yield and quality. A small number of PM-resistant breeding lines and cultivars have been reported in *Gerbera*, but the underlying genetics for PM resistance in *Gerbera* is largely unknown. Scarcity of genomic resources such as genetic linkage maps and molecular markers has severely hindered the effort to understand the genetic basis and locate loci controlling PM resistance in *Gerbera*. This study aimed to construct a genome-wide genetic linkage map, identify quantitative trait loci (QTL), and molecular markers for PM resistance in *Gerbera*. A segregating mapping population was developed by crossing PM-resistant and -susceptible *Gerbera* breeding lines, genotyped by sequencing, and phenotyped for PM resistance. A genome-wide genetic linkage map constructed with 791 single polymorphic site (SNP) markers spans 1912.30 cM across 27 linkage groups (LG) and reaches a density of 1 marker per 2.42 cM. One major consistent QTL was discovered in LG16, explaining more than 16.6% of the phenotypic variance for PM resistance. The QTL was tagged with two flanking SNP markers. The availability of this genetic linkage map will be very useful for locating and tagging QTLs for other important traits in *Gerbera*, and the newly discovered QTL and SNP markers will enable development of molecular markers for improving *Gerbera* for resistance to PM.

## 1 Introduction

*Gerbera* daisy (*Gerbera hybrida*) (2*n* = 2*×* = 50) is commonly grown in many countries as cut flowers, garden flowers, and flowering potted plants. The sales value of cut *Gerbera*s sold by Royal FloraHolland in 2020 alone was €130 million (~$147 million) (https://www.statista.com/statistics/829413/sales-value-of-cut-flowers-sold-by-royal-floraholland-by-type/). Cut *Gerbera*s in the U.S. generated a wholesale value of $32 million in 2015 (USDA, 2016). The continuous introduction of new *Gerbera* cultivars with improved and novel traits is important for the global floral industry. Development of new cultivars in *Gerbera* has predominantly relied on conventional breeding (Deng and Bhattarai, 2018). Increased breeding efficiency is much needed to meet the demands of the floral industry and consumers of new *Gerbera* cultivars with better disease resistance.

There have been few efforts to develop genomic resources in *Gerbera*, including generation of expressed sequence tags, identification of single nucleotide polymorphisms (SNPs), genetic mapping of quantitative trait loci (QTLs), and simple sequence repeats (SSRs) (Gong and Deng, 2010; de Pinho Benemann et al., 2012; Gong and Deng, 2012; Song and Deng, 2013; Fu et al., 2016; Fu et al., 2017). With the advent of next generation sequencing (NGS) and genotyping-by-sequencing (GBS), large numbers of polymorphic sites, such as SNPs, can be readily identified, and construction of genetic linkage maps has become easier in plants for which reference genomes are not available (Elshire et al., 2011; Poland et al., 2012). SNPs are desirable among molecular markers because of their large numbers, ubiquitous distribution, biallelic nature, and high heritability (The International SNP Map Working Group, 2001). GBS is a genome complexity reduction approach that allows sequencing, discovery, and genotyping of thousands of SNPs using restriction enzyme-digested fragments from relatively small amounts of genomic DNA (Ansorge, 2009; Gore et al., 2009). Given the low cost of the technology, GBS has been widely used in several ornamental crops including rose, chrysanthemum, and hydrangea (Horst, 1990; Heo et al., 2017; Song et al., 2020; Wu and Alexander, 2020). In *Gerbera*, genetic analysis had not been done before using genome-wide SNPs and NGS technology. Previously, a linkage map was constructed using SNPs identified from ESTs of *Gerbera* lines segregating for *Botrytis* resistance (Fu et al., 2017). High-density genetic linkage maps are important for plant breeding and genetic research, particularly in plant species for which there are no well annotated reference genomes available. High-density linkage maps can be useful for identifying genes of interest, associating molecular markers with important traits, locating QTLs, and molecular cloning of valuable genes for breeding and genetic studies.

Powdery mildew (PM) is one of the most devastating pathogens infecting many row crops, vegetables, fruits, and ornamental/specialty crops worldwide. Numerous ornamental crops including *Gerbera*, rose, hydrangea, dahlia, and poinsettia can be severely infected by PM in the absence of resistant cultivars (Price, 1970; Benson et al., 2002; Braun et al., 2002; Deng and Bhattarai, 2018; Li et al., 2018). PM, caused by *Podosphaera xanthii* (syn. *Sphaerotheca fusca*), is one of the most challenging diseases in *Gerbera* production (Hausbeck, 2004; Channel, 2005; Granke et al., 2012). Negative impacts of PM in *Gerbera* are observed when the white powder-like spores and mycelia on the flower and plant canopy surfaces develop and severely reduce the aesthetic value. Reduction in visual appeal can result in significant losses to both *Gerbera* nurseries and the cutflower industry. In addition, reduced flower production and stunted plant growth are also caused by PM in controlled and field growing conditions. Various management practices, including the use of fungicides (e.g., azoxystrobin, myclobutanil, and pyraclostrobin), biofungicides (e.g., K-Phite, Millstop, and Tricon), treating with acidic electrolyzed oxidizing water, and burning elemental sulfur have offered preventative or limited post-infection control (Mueller et al., 2003).

Host resistance is considered to be one of the most effective, environmentally friendly and cost-effective methods to control PM and minimize crop losses in *Gerbera*. Developing PM-resistant *Gerbera* cultivars could offer a long-term solution to mitigate the disease, reduce the use of fungicides, and lower costs in *Gerbera* production. A better understanding of the genetics underlying PM resistance in *Gerbera* will facilitate the development of new PM-resistant cultivars. The main objectives of this study were to i) construct a genome-wide genetic linkage map using a biparental segregating mapping population and high-quality SNPs discovered from genotyping by sequencing and ii) identify QTLs and markers for PM resistance in *Gerbera*. This study is the first instance in *Gerbera* where GBS was used to discover SNPs and construct genetic linkage maps. Thus, the results from this study fill a major knowledge gap in *Gerbera* genetics and will likely advance PM resistance breeding efforts in *Gerbera*.

## 2 Results

### 2.1 Restriction enzyme combination and sequencing depth on SNP discovery

**Figure 1:**
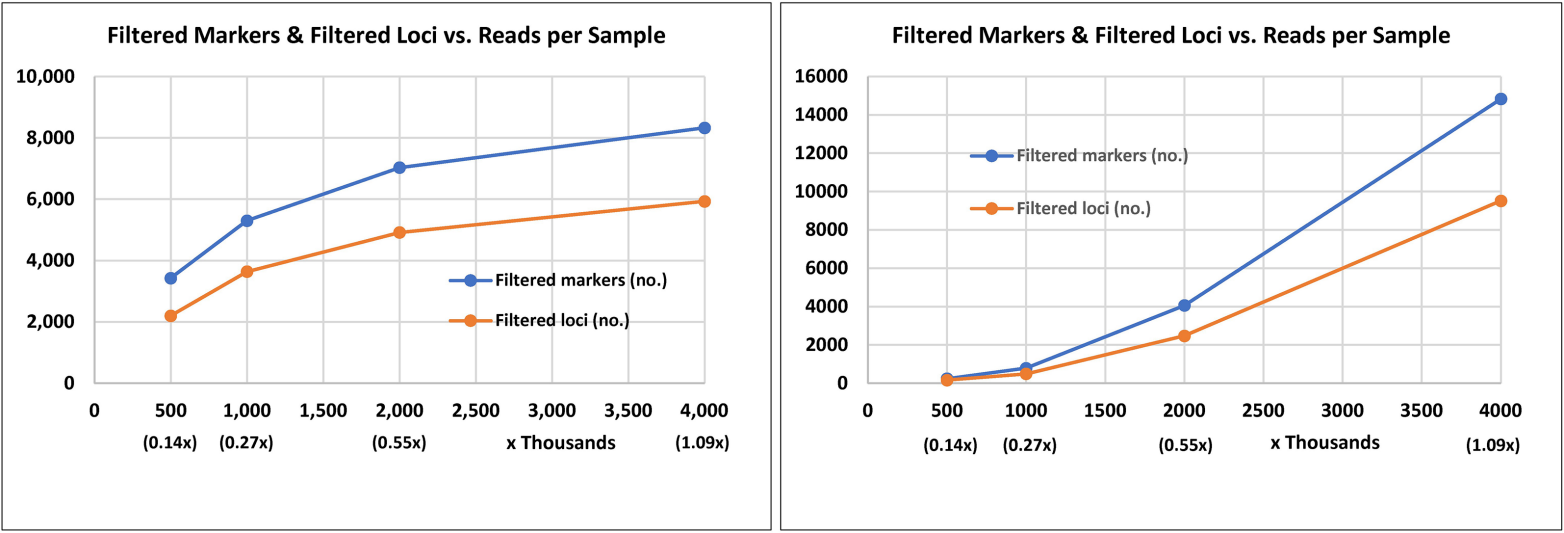
Marker and locus discovery rate from genotyping by sequence after digestion of *Gerbera* genomic DNA with two combinations of restriction enzymes and sequencing at different read depths (number of reads per sample). The combination of *Bam*HI and *Nsi* was used in the left chart and the combination of *Btg*I and *Taq*I in the right chart. Genomic DNA digestion, sequencing library preparation, and genotyping by sequencing were completed at the University of Minnesota Genomics Center.

Two GBS experiments were conducted to select restriction enzymes (REs) and determine the sequencing depth for discovering SNPs in *Gerbera*. These experiments consisted of both parental lines, one pool of PM-resistant progenies, and one pool of PM-susceptible progenies. The number of loci and the total number of SNPs discovered in the two GBS experiments are shown in **Figure 1** and **Supplementary Table 1**. Digestion of genomic DNA with *Bam*HI and *Nsi*I produced 7,029 SNPs when sequenced at 2 million reads/sample as compared to 4,059 SNPs when sequenced at 2 million reads/sample using *Btg*I and *Taq*I. Although, digestion of *Gerbera* genomic DNA with *Btg*I and *Taq*I prior to GBS generated a greater number of SNPs (14,826) when compared to digestion with *Bam*HI and *Nsi*I, which generated 8,327 SNPs when each sample was sequenced with 4 million reads, the rate of increment of SNP discovery declined as the reads per sample increased from 2 million to 4 million per sample (**Figure 1**). Digestion with *Bam*HI and *Nsi*I and sequencing rate of 2 million reads per sample seemed adequate for genotyping the *Gerbera* population in this study.

### 2.2 Linkage map construction

**Figure 2:**
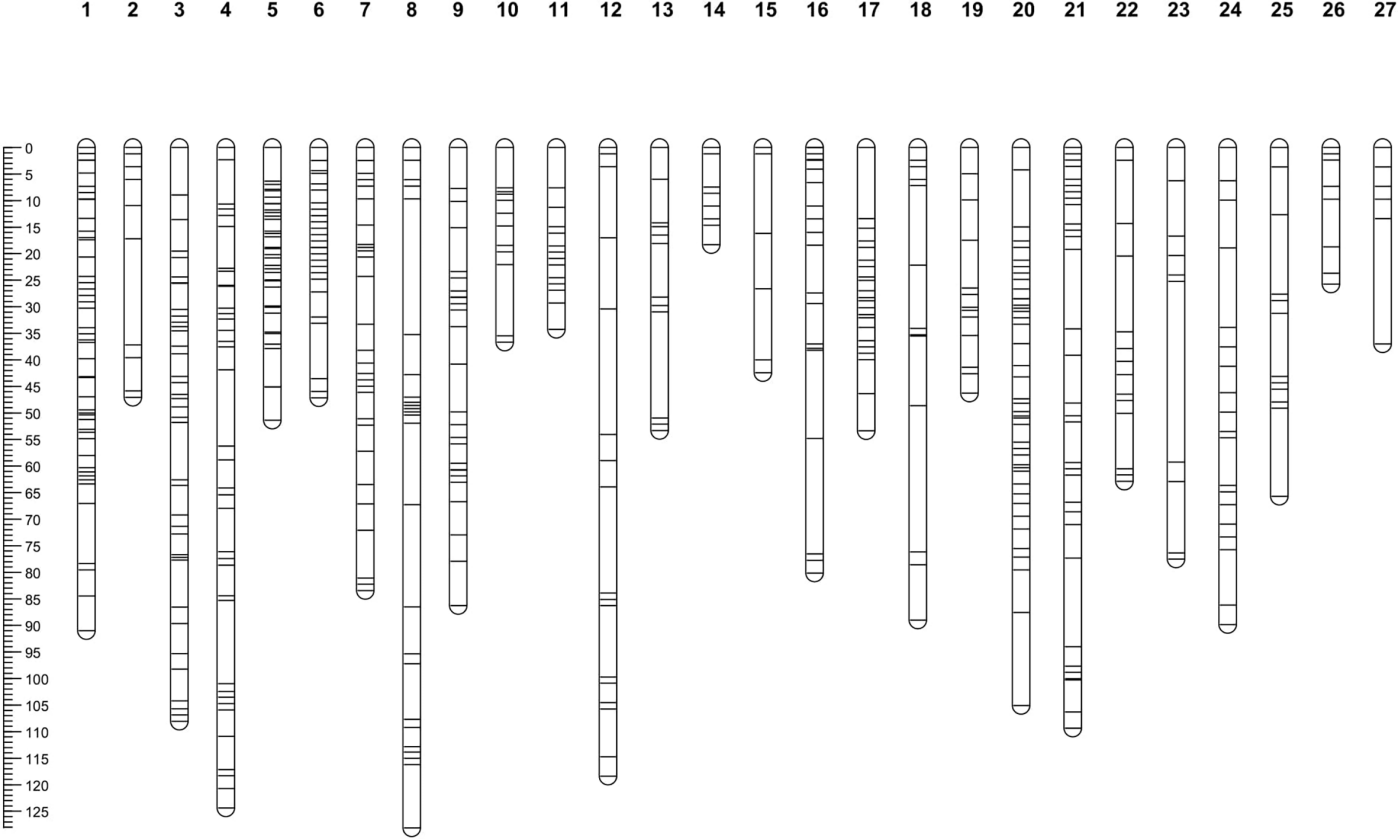
Linkage map of *Gerbera hybrida* containing 27 linkage groups developed by using SNPs discovered from genotyping by sequencing. The mapping F_1_ population consisted of 88 individuals developed by crossing two breeding lines UFGE 4033 and 06-245-03. The scale plate on the left displays genetic distance in centiMorgan (cM) that was calculated using the Kosambi mapping function.

In total, 88 segregating individuals were genotyped-by-sequencing using the *Bam*HI and *Nsi*I for genomic DNA digestion. Three individuals were removed from further analyses because their GBS data failed to meet the SNP filtering criteria. In total, 3,541 SNPs and the genotyping data from 85 progenies were used in JoinMap 4.0 (Van Ooijen, 2006) for the linkage map construction. SNPs that showed recombination frequencies (> 0.4), distorted segregation ratios (χ^2^ > 11.31; *p <* 0.0001), or high nearest-neighbor stress values (> 10 cM) were excluded from use for the map construction. Out of 3,541 SNPs, 791 SNPs were mapped in the linkage map. Among 791 SNPs, there were 277 (35%) SNPs that were heterozygous in the maternal parent and homozygous in the paternal parent and segregated in the expected ratio of 1:1 ‘lm × ll’, 439 (55%) SNPs heterozygous in the paternal parent and homozygous in the maternal parent and segregated in the expected ratio of 1:1 ‘nn × np’, and 75 (9%) SNPs were heterozygous in both parents and segregated in the expected ratio of 1:2:1 ‘hk × hk’, respectively. These data indicated that 06-245-03 carried a greater number of heterozygous loci compared to UFGE 4033. In total, 791 SNP markers were mapped to 27 linkage groups (LGs) (**Figure 2**). The description of LGs including distance and number of SNPs are shown in **Table 1**. The overall average length of the LGs was 70.83 cM. The composite genetic linkage map spans 1912.30 cM with an average of 2.42 cM between SNP markers. The largest LG was LG04 spanning 124.4 cM, and the smallest was LG14 with a genetic distance of 18.3 cM. LG01 contains the largest number of SNPs (62), whereas LG15 and LG27 contain the smallest number of SNPs (6). There were 12 gaps in the linkage groups that each span more than 15 cM, with an average genetic distance of 15.17 cM between two adjacent markers. These gaps are present in 12 linkage groups (LG02, LG04, LG08, LG12, LG13, LG15, LG16, LG18, LG20, LG21, LG23, and LG27). The largest gap is in LG18, which extends 27.54 cM (**Table 1**).

**Table 1:** Description of *Gerbera* genetic linkage groups constructed using SNPs discovered by genotyping by sequencing of a biparental mapping population (F_1_).

Linkage Group	Number of markers mapped	Length (cM)	Marker density (markers per cM)	Largest gaps between markers (cM)
LG01	62	91.00	0.68	11.32
LG02	13	47.10	0.28	19.98
LG03	60	108.10	0.56	10.82
LG04	59	124.40	0.47	15.68
LG05	55	51.40	1.07	7.21
LG06	38	47.20	0.81	10.43
LG07	42	83.50	0.50	9.00
LG08	37	128.10	0.29	25.54
LG09	39	86.30	0.45	8.33
LG10	20	36.70	0.54	13.41
LG11	18	34.20	0.53	7.11
LG12	22	118.40	0.19	19.80
LG13	19	53.30	0.36	19.98
LG14	13	18.30	0.71	5.91
LG15	6	42.40	0.14	15.00
LG16	38	80.20	0.47	21.77
LG17	31	53.40	0.58	13.42
LG18	21	89.00	0.24	27.54
LG19	17	46.20	0.37	5.98
LG20	56	105.10	0.53	17.50
LG21	43	109.40	0.39	16.68
LG22	19	62.90	0.30	14.29
LG23	10	77.50	0.13	34.10
LG24	26	89.80	0.29	13.30
LG25	13	65.70	0.20	16.60
LG26	8	25.70	0.31	9.00
LG27	6	37.00	0.16	19.80
Average		70.83	0.41	15.17
Total	791	1912.30		

### 2.3 Phenotyping and segregation of PM resistance

**Figure 3:**
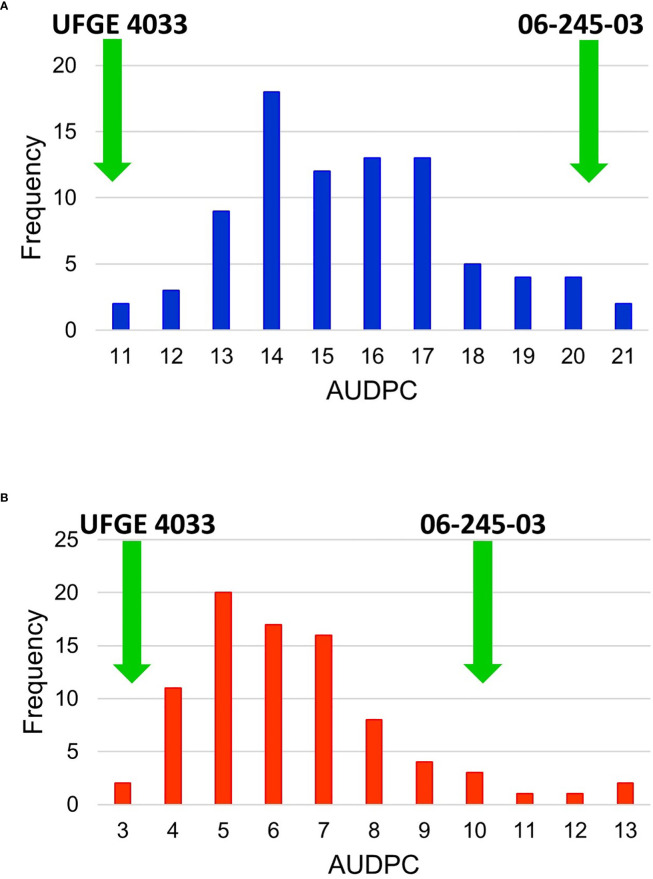
Frequency histogram of the Area Under Disease Progress Curve (AUDPC) values of an F_1_ segregating population in **(A)** year 2018 and **(B)** 2019 for powdery mildew. The segregating population was developed by crossing *Gerbera* breeding lines 06-245-03 and UFGE 4033. The green arrows indicate the scores of resistant (UFGE 4033) and susceptible (06-245-03) parents used in developing the mapping population.

All progenies in the mapping population (and both parents) were phenotyped for PM resistance inside a greenhouse over two years (2018-2019). Each progeny was asexually propagated to obtain four clonal plants, which served as four biological replicates. Visual score rating was performed by evaluating the presence of PM spores and mycelial growth on the plant surfaces. Each disease evaluation was performed every seven days for four consecutive weeks. Area under disease progress curve (AUDPC) scores for each year for every individual were calculated separately. No significant block (or replicate) effect was observed, therefore the average of all evaluations within a year was calculated and used for QTL mapping (**Supplementary Table 2**). Histograms of the mapping population with the frequency of individuals in each AUDPC category for 2018 and 2019 are shown in **Figure 3**. The AUDPC score for individuals ranged from 11.17 to 21.63 and averaged 16.00 in 2018; the AUDPC score for individuals ranged from 3.50 to 13.25 and averaged 6.90 in 2019. The AUDPC scores for the PM-resistant parent UFGE 4033 in 2018 and 2019 were 11.17 and 3.23, respectively, whereas the AUDPC scores for the PM-susceptible parent 06-245-03 breeding line in 2018 and 2019 were 20.36 and 10.18, respectively. Based on the frequency distribution of the disease severity in the mapping population, the histogram showed an approximately normal distribution, and therefore, no data transformation was performed prior to QTL analysis. The correlation coefficient of the AUDPC scores of the mapping population in 2018 and 2019 was 0.60.

### 2.4 QTL analysis for PM resistance

**Figure 4:**
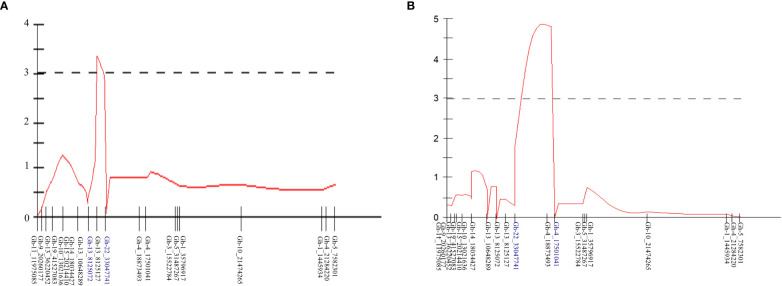
QTLs for powdery mildew (PM) resistance identified in **(A)** 2018 and **(B)** 2019 using the phenotypic PM scores and linkage map developed using F_1_ segregating mapping population by crossing *Gerbera* breeding lines UFGE 4033 (resistant to PM) and o6-245-03 (susceptible to PM).

QTL analysis was performed for the PM AUDPC scores for 2018 and 2019 separately. The newly constructed linkage map containing 791 SNPs and the yearly AUDPC values from individuals in the mapping population calculated for each season were used in the MapQTL program for QTL analysis. Initially, interval mapping was performed and the LOD score was determined by permutation 1,000 tests. For year-wise QTL analysis, a LOD score significant at the level of *p < 0.05* was used to determine the significance of the QTLs identified. MQM analysis was performed by assigning the significant markers in the proximity of the identified locus. Based on the phenotypic PM AUDPC values in 2018, one QTL was identified in LG16 and the associated marker, Gh-13_8125127, located at 15.98 cM with a LOD of 3.35 (**Table 2**; **Figure 4**). This QTL explained 16.6% of the phenotypic variation in 2018. Based on the AUDPC values in 2019, the same procedure identified one QTL in the same linkage group, LG16, and the marker associated with the locus is Gh-4_18873493 located at 27.41 cM. The locus was significant with a LOD score of 4.83. This locus explained 20.4% of the phenotypic variance in 2019 (**Table 2**). This locus was identified in both years and hence was considered consistent.

**Table 2:** QTLs identified by using the linkage map and *Gerbera* powdery mildew resistance phenotyping data from 2018 and 2019.

Year	Linkage group	Position(cM from the end)	Locus	QTL interval (cM)	LOD	% phenotypic variance
2018	LG16	15.99	Gh-13_8125127	4.94	3.35	16.6
2019	LG16	27.41	Gh-4_18873493	11.00	4.83	20.4

## 3 Discussion

In this study, GBS was used to identify SNPs using a biparental population developed by crossing two *Gerbera* breeding lines. Two small pooled GBS experiments conducted on parental lines and resistant and susceptible pools confirmed the successful use of sequencing depth of 2 million reads per sample and selection of *Bam*HI and *Nsi*I REs for population genotyping and obtaining required number of SNPs for linkage mapping and QTL analysis. In the NGS-era, SNP markers have been widely used for linkage map development because of their abundance and distribution throughout plant genomes (Pootakham et al., 2015; Verma et al., 2015; Yang et al., 2017). GBS facilitates the discovery and genotyping of large numbers of novel SNPs distributed from telomere to telomere in multiple samples simultaneously. Identification of large number of SNPs increases the flexibility of retaining a higher density of SNPs per cM, even after removing SNPs that do not pass all filtering steps and fit in the linkage maps. GBS has been used to construct high-density linkage maps in a wide range of herbs, trees, vegetables, fruits, and ornamental crops including chickpea, rubber tree, pea, maize, sweet cherry, wheat, rose, and sunflower (Leonforte et al., 2013; Guajardo et al., 2015; Li et al., 2015; Pootakham et al., 2015; Verma et al., 2015; Celik et al., 2016; Su et al., 2017; Li et al., 2019). In this study, we show that GBS can be used in *Gerbera* to identify SNPs and perform linkage mapping.

Construction of genetic linkage maps has been reported in few ornamental crops including rose, carnation, sunflower, tulip and lotus (Debener and Mattiesch, 1999; Hayashi et al., 2001; Han et al., 2002; Yu et al., 2003; Yagi et al., 2013; Tang et al., 2015). The large genome size, large number of chromosomes (2*n* = 50), and limited availability of molecular markers have hindered the construction of linkage map in *Gerbera*. In this study, we constructed a genetic linkage map by using GBS-generated SNPs and a F_1_ biparental population. In outcrossing ornamental crops, most studies have used F_1_ populations to construct linkage maps (Debener and Mattiesch, 1999; Rajapakse et al., 2001; Han et al., 2002; Shahin et al., 2011; Zhang et al., 2015; Fu et al., 2016). Use of segregating F_1_ populations was due to occurrence of severe inbreeding depression and no seed formation or seed decline as homozygosity increases with the advancement of generations *via* selfing. Prior to this study, one linkage map was available in *Gerbera* which was developed based on markers discovered from ESTs and an F_1_ population (Fu et al., 2016). As the first GBS study in *Gerbera*, we observed interesting phenomena in this species. Out of the high-quality SNPs from GBS, only 22.3% of them could be mapped to the linkage groups. Many SNPs showed significant segregation distortion ratios, unexpected high recombination frequencies, or high nearest-neighbor stress values. These phenomena might have resulted from *Gerbera*’s highly heterozygous, complex genome and/or the lack of high-quality reference genome for reliable variant calls. Although the cause(s) for this high portion (nearly 78%) of unmappable SNPs remain(s) to be understood, it does suggest the need for discovery of a lot of more SNPs for genetic mapping in *Gerbera*.

Our new *Gerbera* linkage map has an average distance of 2.42 cM between markers. Compared to the previous *Gerbera* linkage map, this linkage map has a better genome coverage, fewer gaps (12) that are > 15 cM, a higher marker density, and a larger number of SNPs. These improvements are due to the use of genome-wide high-quality SNPs. Due to the increased coverage of the genome by the linkage map constructed in this study, it could be useful to perform QTL studies, develop molecular markers and assemble chromosomes during genome assembly and construction of physical maps. The current linkage map consisted of 27 LGs, which was two more than the expected 25 chromosomal level-LGs given that *Gerbera* is a diploid with 2*n* = 50 chromosomes. This result suggests the presence of two large gaps each of which is bigger than 15 cM in one or two of the LGs obtained in this study, accounting for the two extra LGs. Gaps present in the current linkage map could be potentially minimized by increasing the genome coverage during sequencing leading to increased SNPs discovery. Another strategy for gap reduction could be use of larger mapping populations, multiple crossing families and higher marker densities to capture more recombination events to merge the broken linkage groups.

In this study, we relied on the naturally available PM conidia in the environment (greenhouse) as inoculum to infect the F_1_ mapping population. In Florida’s warm and humid climate, PM can survive as mycelium and conidia, reproduce asexually and continuously cause disease as *Gerbera* can be grown year-round in both landscape and greenhouse conditions. Indeed, PM infection was observed year-round on the *Gerbera* plants in our greenhouse previously and during this study. Prior to this study, Kloos et al. (2005) assessed PM incidences and severity on *Gerbera* based on natural inoculation in the greenhouse and field studies (Kloos et al., 2005). The disease severity distribution under the two inoculation schemes was similar (Song and Deng, 2013). The fact that our PM phenotypic data from 2018 and 2019 allowed us to independently map two QTLs in the same linkage group (LG16; despite being ~11cM apart) attested the adequacy of natural PM infection of the F_1_ population in our greenhouse. However, inoculation of *Gerbera* plants with a pure PM isolate, when available in the future, should further increase the accuracy of phenotyping results, which in turn should increase the accuracy of the map location of the QTL contributing to PM resistance in UFGE 4033.

The PM fungal development in resistant and susceptible *Gerbera* has been previously studied (Song and Deng, 2013). When the conidia land and establish on the leaf surfaces, they germinate in both resistant and susceptible *Gerbera* lines. However, hyphal branching was observed in the susceptible lines whereas it was significantly reduced in the resistant lines at 72 hours post inoculation (Song and Deng, 2013). Similar reduction of haustorial and hyphal growths have been observed on the leaf surface of PM resistant line in cucumber, PI197088-1 (Hausbeck et al., 2002). Previous genetic studies suggested that PM resistance in *Gerbera* may be attributed to a single gene and/or QTL (Kloos et al., 2005). Kloos et al. reported that although a single dominant gene *Pmr1* (powdery mildew resistance 1) was shown to confer the resistance in *Gerbera*, *Pmr1-*controlled resistance appeared to be partially relied on other unidentified genes that may modify the effect of *Pmr1* (Kloos et al., 2005). Multiple PM resistance mechanisms in cucumber are known to confer the resistance including recessive genes showing *R*-gene like response including HR-like spots (Sakata et al., 2006), accumulation of serine/threonine-protein kinase receptor (RPK2) (Zhang et al., 2021) or loss of function of *CsMLO1* susceptibility gene (Nie et al., 2015). However, the molecular mechanisms underlying the resistance in both *Gerbera* and cucumber remain to be completely understood. Our previous study by Song and Deng using a limited number of markers designed from ESTs suggested that the PM resistance in *Gerbera* to be a quantitative trait controlled by one or more major genes (Song and Deng, 2013). In that study using UFGE 4033 and a PM-susceptible line Sunburst Snow White, two QTLs, resistance to *Podosphaera xanthii* 1 (*Rpx1*) and *Rpx2* were identified in UFGE 4033 to confer resistance to PM (Song and Deng, 2013). These QTLs contributed more than 71% of the resistance and *Rpx1* alone explained 56.5% PM resistance (Song and Deng, 2013). However, this study only included 17 EST-SSR markers and the QTLs could not be assigned to specific linkage group or chromosome. In the present study, we used genome-wide distributed SNPs which, theoretically, offers comprehensive search and analysis of QTLs present in *Gerbera* for PM resistance. Interestingly, we identified a QTL locus on LG16 using both the 2018 and 2019 phenotypic data spanning 4.95 cM and 11.00 cM and explaining 16.6% and 20.4% of phenotypic variance, respectively. This locus was tentatively named as *Gerbera hybrida* powdery mildew resistance locus 1 (*GhPMR1*). Some of the linked SNPs have been converted into high-resolution melting curve (HRM) markers (**Supplementary Figure 1**). They are or will be used for fingerprinting *Gerbera* lines and selecting proper F_1_ individuals to create new segregating populations for identifying additional PM resistance QTLs.

Disease severity in year 2018 was higher compared to that in year 2019. High disease pressure in year 2018 could have revealed a locus with a sharper LOD peak whereas low disease pressure in year 2019 might have ensured more accurate determination of the disease infection phenotypes of the F_1_ population, resulting in a more confined map location of the QTL. Disease distribution in the mapping population and phenotypic variance explained by *GhPMR1* demonstrate that PM resistance in *Gerbera* is controlled by more than one locus. As observed in other flowers or closely related species including sunflower, lettuce, and rose, PM resistance in *Gerbera* is very likely controlled by multiple loci. Low phenotypic variance (16.6% and 20.4% in 2018 and 2019, respectively) explained by the identified QTL could be attributed to the relatively small mapping population size (88 individuals) as compared to a rather large number of linkage groups (25) of *Gerbera*. The small population size also severely limited the resolution of the genetic map. Development of larger mapping populations could increase the recombination events and identification of additional SNPs for filling the gaps in the linkage map. Increased recombination events and SNPs could increase the marker density and the resolution in the genetic map, which in turn should increase the probability of identifying other QTLs and explaining more phenotypic variance for PM resistance. Availability of a high-quality reference genome in future could facilitate the identification of potentially all genomic regions contributing to PM resistance in *Gerbera*.

In summary, this study has used genome-wide SNPs for the first time to construct a genome-wide genetic linkage map and identified a major QTL for PM resistance in *Gerbera*. These results will be useful for developing molecular markers for marker-assisted selection of *Gerbera* for PM resistance, tagging other traits important in *Gerbera*, and identifying the genes present in the QTL regions that control PM resistance in *Gerbera*.

## 4 Materials and methods

### 4.1 Plant materials

A segregating F_1_ population comprising of 88 individuals was developed by crossing two *Gerbera* breeding lines exhibiting PM resistance (UFGE 4033) and susceptibility (06-245-03) as reported in the previous study (Bhattarai et al., 2020). UFGE 4033 was developed at the University of Florida and is resistant to PM (Deng and Harbaugh, 2013; Song and Deng, 2013). UFGE 4033 is characterized by shiny large green leaves, long peduncles, semi-double flower, soft-white ray-florets, and light green disc florets, and it can be grown for both pot and cut flower production. UFGE 4033 consistently showed resistance to PM in both fields and greenhouse screening studies and in the current study (Gong and Deng, 2010; Song and Deng, 2013; Bhattarai et al., 2020). UFGE 4033 has been used in *Gerbera* breeding programs as a source of PM resistance in developing new cultivars (Deng and Bhattarai, 2018). UFGE 4033 originated from a cross between breeding line UFGE 31-19 (PM-resistant) and UFGE 35-4 (PM-susceptible). Hence, the PM resistance in UFGE 4033 was expected to be heterozygous. Contrastingly, 06-245-03 has dark green leaves, short peduncles, simple flowers, dark-brown disc center, and dark red ray florets, and it is primarily grown for pot plant production. Previously, breeding line 06-245-03 was identified as being highly susceptible to PM (Bhattarai et al., 2020).

Artificial crossing between the two breeding lines was done in a greenhouse at the Gulf Coast Research and Education Center in Wimauma, FL, USA from October 2015 to March 2016. Seeds developed from the crosses were sown in September 2016 in plastic 72-cell trays and were kept in a growth room at 25°C with 16/8-hour day-night light conditions. Seedlings were grown for a month until they had three to four true-leaves and then transferred to four-inch plastic containers filled with a soilless potting mixture Faffard^®^ 3B (50% Canadian peat and 50% of the mixture of vermiculite, pine bark and perlite) (Agawam, MA, USA). Seedlings (progeny) were later individually transferred to 3.79-L plastic containers and grown in these containers for six months to develop enough crowns for multiplication. The crown of each plant was divided into four parts of similar sizes and grown individually as four replicates for the progeny for subsequent studies. Subsequently, the clonally propagated plants were grown in two-gallon plastic containers in a greenhouse from 2017 to 2019. As expected, the progeny of this population segregated for PM resistance as well as for various other traits including plant height, peduncle length, flower color, and leaf color (data not shown).

### 4.2 Genotyping by sequencing

Genotyping by sequencing was conducted in two phases. In the first phase, the genomic DNA of two *Gerbera* parents (UFGE 4033 and 06-245-03) in two replicates (four samples total), one pool of PM-resistant individuals, and one pool of PM-susceptible individuals were digested with one of the two combinations of restriction enzymes (*Bam*HI and *Nsi*I, or *Btg*I and *Taq*I) prior to sequencing to evaluate the effect of restriction enzyme combinations on SNP marker discovery. The resistant pool consisted of 10 F_1_ individuals Gh-213, Gh-77, Gh-243, Gh-3, Gh-202, Gh-288, Gh-281, Gh-191, Gh-256, and Gh-212. The susceptible pool consisted of 13 F_1_ individuals Gh-21, Gh-27, Gh-306, Gh-303, Gh-47, Gh-234, Gh-50, Gh-302, Gh-37, Gh-308, Gh-19, Gh-2, and Gh-42. In the second phase, all F_1_ progenies of the mapping population and their two parents were subjected to GBS with *Bam*HI and *Nsi*I as the restriction enzymes for genomic DNA digestion.

Genomic DNA was extracted from young, unopened leaves using a DNeasy Plant Mini Kit (Qiagen, Hilden, Germany) following the manufacturer’s protocol. DNA concentrations were determined on a Nanodrop 8000 spectrophotometer (Fisher Scientific, Waltham, MA, USA) and a Qubit 4 fluorometer (Thermo Fisher Scientific, Waltham, MA, USA) using the Quant-iT™ PicoGreen^®^ (Thermo Fisher Scientific). DNA preparations with the concentration >10 ng/µL and a minimum quantity of 200 ng were used for GBS library preparation.

Library preparation and sequencing for GBS were conducted at the University of Minnesota Genomics Center (Minneapolis, MN). Briefly, genomic DNA was digested with a combination of two restriction enzymes, followed by ligation of adapters consisting of barcode sequences and Illumina sequencing primers. The ligated DNA fragments from all samples were pooled, PCR-amplified, and sequenced in one Flowcell on a NextSeq 500 sequencer (Illumina, San Diego, CA, USA). Sequencing was performed using the single end chemistry with a read length of 150 bases.

### 4.3 Variant calling and SNP analysis

Raw sequencing data were converted to de-multiplexed Fastq files using the Illumina bcl2fastq software. Adapters at the beginning and the end of the raw reads were removed using Trimmomatic (Bolger et al., 2014). The resultant Fastq files were aligned to a draft partial *Gerbera* genome sequence (developed using the Illumina and PacBio sequencing approaches for the program’s internal use) (Bhattarai and Deng, unpublished data) using the Burrows-Wheeler Alignment tool (Li, 2013). Freebayes was used to jointly call variants across all samples simultaneously (Garrison and Marth, 2012). The raw VCF file generated by Freebayes was filtered using the VCFtools to remove variants with the minor allele frequency < 1%, variants with genotype rates < 95%, and samples with genotype rates of < 50% (Danecek et al., 2011).

### 4.4 Linkage map construction

A linkage map was constructed using SNPs discovered from the mapping population. SNPs identified from the variant calling procedure were filtered to select high-quality SNPs. SNPs that were not called and absent in more than 8 individuals (10% of the population) were removed from further analysis. Individuals missing more than 2% genotypic data were excluded. Chi-square tests were performed to detect potential segregation distortion for each SNP, and those SNPs with *χ^2^* > 11.31 (*p <* 0.0001*)* were discarded. Additional SNPs were excluded due to unexpected high recombination frequencies (> 0.40) or high nearest-neighbor stress values (> 10 cM). The *Gerbera* segregating population type was coded as cross pollinated (CP), and the genotype for SNPs were coded “hk × hk” when both parents were heterozygous, “nn × np” when parent 1 (P_1_) was homozygous and P_2_ was heterozygous, and “lm × ll” when P_1_ was heterozygous and P_2_ was homozygous for the given locus. Linkage groups (LGs) were constructed using the JOINMAP^®^ 4.1 program with the following parameters: A minimum logarithm of odds (LOD) score of 5, a maximum recombination frequency of 0.40, and goodness-of-fit jump at 5 (Van Ooijen, 2006). The maximum likelihood algorithm and the Kosambi mapping function with 3 reiterations were selected for construction of the linkage map. The linkage groups were drawn using MapChart (Voorrips, 2002).

### 4.5 Phenotyping PM resistance

Individuals of the segregating population and its parents were evaluated for PM resistance in a greenhouse. All plants were arranged in four blocks on metal benches. Each block contained one replicate of all individuals and the parents randomly placed on the metal benches. The individuals were exposed to natural PM infection in the greenhouse. The greenhouse had been used to grow *Gerbera* plants for more than 10 years, and severe PM was observed in the greenhouse during this period. To promote PM development in the greenhouse, two highly susceptible commercial cultivars, Tangerine Dark Eye and Yellow Dark Eye, were grown and placed among the *Gerbera* individuals. PM disease scoring began 30 to 45 days after moving *Gerbera* plants into the greenhouse or repotting of the plants. Uniform distribution of the inoculum in the whole greenhouse was maintained by running circulation fans within the greenhouse. These actively growing foliar parts had equal chances for freely spreading PM spores to land and germinate on the foliar leaf surfaces. All individuals were phenotyped over two years in 2018 and 2019 to capture the variation caused due to difference in growing conditions. PM severity was rated using a scale of 1 to 10 as described previously with minor modifications: 1 = no disease, 2 = trace to 10%, 3 = 10 to 20%, 4 = 20 to 30%, 5 = 30 to 40%, 6 = 40 to 50%, 7 = 50 to 60%, 8 = 60 to 70%, 9 = 70 to 80%, and 10 = 80 to 100% (Hausbeck et al., 2002). The disease severity data were recorded for four weeks and following a weekly disease rating procedure. The means of the four replications were calculated and used for Area Under Disease Progress Curve (AUDPC) calculation as described previously (Jeger and Viljanen-Rollinson, 2001). AUDPC scores for each individual and for each season were used in QTL analysis.

### 4.6 Quantitative trait loci analysis

QTL mapping was performed by analyzing genotyping data of the mapped SNPs, and the disease severity of the mapping population using mapQTL^®^ 6 (Van Ooijen, 2009). Putative QTL regions associated with PM resistance were detected in the LGs using interval mapping. After performing interval mapping, MQM was performed by assigning the closest markers as co-factors. Determination of LOD score threshold was performed by 1,000 permutation tests corresponding to a genome-wide confidence level of P < 0.05. The software also estimated the percentage of phenotypic variance and additive effect explained by a QTL for a trait. QTL analysis was performed for 2018 and 2019 individually and the loci appearing in both years were considered consistent.

## 5 Conclusion

A genome-wide genetic linkage map was constructed for *Gerbera* using SNPs discovered by GBS. The linkage map contained 791 SNPs distributed across 27 LGs and spanning 1912.30 cM. A QTL, *GPhMR1* conferring resistance to PM, was discovered in LG16. *GPhMR1* explained more than 16.6% phenotypic variance for PM resistance. The locus was tagged with two markers Gh-13_8125127 and Gh-4_18873493 located at 15.987 cM and 27.410 cM, respectively. This locus and the SNP markers will be useful for breeding *Gerbera* for PM resistance in future. Similarly, the genetic linkage map developed could be utilized in studying genetic architecture of other traits, identification of novel QTLs, and developing molecular markers associated with those traits in *Gerbera*, one of the top cut flowers in the world.

## Data availability statement

The Gerbera genotyping by sequencing data presented in the study are deposited in the NCBI BioProject: PRJNA902916, https://www.ncbi.nlm.nih.gov/bioproject/PRJNA902916.

## Author contributions

ZD designed and supervised the project, secured the funding, and critically revised the manuscript; KB performed the experiments, analyzed data, and wrote the manuscript; SS assisted in population development, plant propagation, and disease phenotyping; SV guided the linkage mapping analysis; NP, SX, and DC provided essential guidance to the study and provided essential comments and critically revised the manuscript. All authors contributed to the article and approved the submitted version.

## Supplementary material

Supplementary Figure 1 Histogram of one high-resolution melting (HMR) curve marker developed from one of the SNPs that is linked to the major QTL *GhPMR1*. Two patterns, indicated by the red and blue melting curves, were evident among the *Gerbera* parental and F_1_ individuals. This marker could clearly differentiate *Gerbera* individuals, but as the QTL *GhPMR1* explained only 16.6% or 20.4% of the phenotypic variance for PM resistance, this marker had only a limited diagnostic capability for PM resistance. Additional QTLs and SNP markers are needed for reliable selection of PM-resistant *Gerbera* breeding lines.
