# Supplementary figures and images for: Construction of a genome-wide genetic linkage map and identification of quantitative trait loci for powdery mildew resistance in Gerbera daisy
Source: Front Plant Sci. 2023 Jan 6;13:1072717. doi: 10.3389/fpls.2022.1072717 (PMC9853552; doi:10.3389/fpls.2022.1072717)

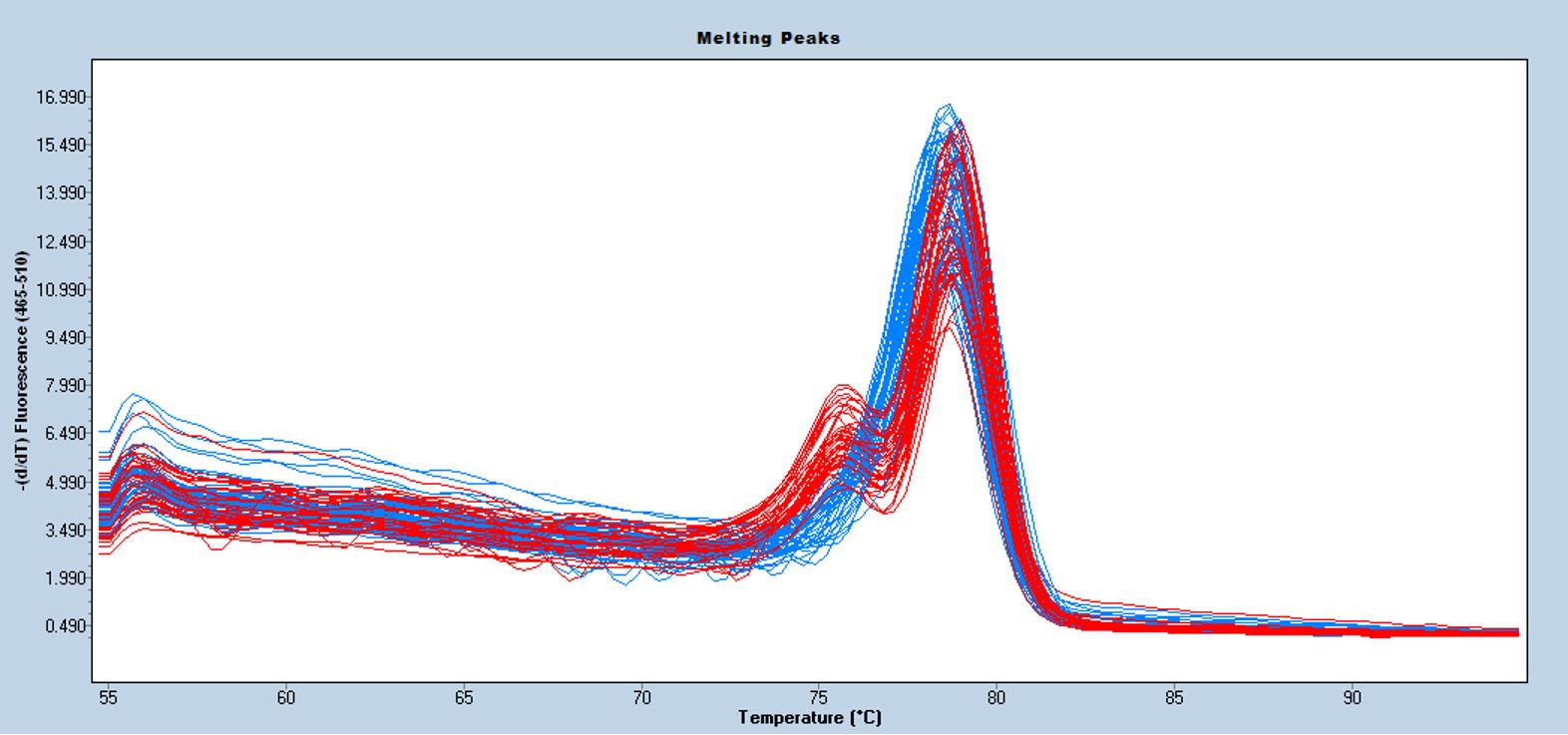

Supplement: Supplementary Figure 1 — Histogram of one high-resolution melting (HMR) curve marker developed from one of the SNPs that is linked to the major QTL GhPMR1. Two patterns, indicated by the red and blue melting curves, were evident among the Gerbera parental and F1 individuals. This marker could clearly differentiate Gerbera individuals, but as the QTL GhPMR1 explained only 16.6% or 20.4% of the phenotypic variance for PM resistance, this marker had only a limited diagnostic capability for PM resistance. Additional QTLs and SNP markers are needed for reliable selection of PM-resistant Gerbera breeding lines. [file Image_1.jpeg]
